# DEPTOR has growth suppression activity against pancreatic cancer cells

**DOI:** 10.18632/oncotarget.2659

**Published:** 2014-12-30

**Authors:** Hua Li, Grace Y. Sun, Yongchao Zhao, Dafydd Thomas, Joel K. Greenson, Mark M. Zalupski, Edgar Ben-Josef, Yi Sun

**Affiliations:** ^1^ Division of Radiation and Cancer Biology, Department of Radiation Oncology, University of Michigan, Ann Arbor, MI 48109, USA; ^2^ Department of Pathology, University of Michigan, Ann Arbor, MI 48109, USA; ^3^ Department of Internal Medicine, University of Michigan, Ann Arbor, MI 48109, USA; ^4^ Institute of Translational Medicine, Zhejiang University School of Medicine, Hangzhou, Zhejiang, P.R. China

**Keywords:** DEPTOR, mTOR pathway, pancreatic cancer, growth suppression

## Abstract

DEPTOR was reported as a naturally occurring inhibitor of mTORC1 and mTORC2. The role of DEPTOR in the growth and survival of pancreatic cancer cells has not previously been determined. Here we report that while DEPTOR shows a cytoplasmic expression in both normal pancreatic acinar and islet cells in a patchy manner, its expression is reduced in PanIN1 and PanIN2 and completely lost in 100 out of 101 pancreatic ductal adenocarcinoma (PDAC) tissues. Ectopic DEPTOR expression in two pancreatic cancer cell lines, Panc-1 and Miapaca-2, caused a significant 1) suppression of anchorage-dependent growth in monolayer culture, particularly under conditions with growth factor deprivation; 2) decreased clonogenic survival, and 3) suppressed anchorage-independent growth in soft agar. These effects are attributable to moderate induction of apoptosis and growth arrest at the S and G2/M phases, in a cell line dependent manner. Furthermore, ectopic DEPTOR expression moderately inhibited mTORC1 activity, as demonstrated by reduced phosphorylation of S6K, S6, and 4E-BP1. Taken together, these data suggest that DEPTOR has a tumor suppressive activity against pancreatic cancer cells, and its loss of expression may contribute to pancreatic tumorigenesis.

## INTRODUCTION

DEPTOR is a recently identified mTOR binding protein that inhibits both mTORC1 and mTORC2 [1], the central regulators of cell metabolism, growth, proliferation, and survival [2–4]. In cell culture settings, DEPTOR mainly acts as a tumor suppressor, since DEPTOR loss activates mTORC1 and mTORC2 and promotes growth and survival of multiple cancer cell lines. However, in a subset of multiple myeloma cells, where DEPTOR was overexpressed, DEPTOR acts as an oncogene and survival factor, since DEPTOR inhibition of mTORC1 relieves the feedback inhibition from S6K1 to PI3K, boosting AKT activity for cancer cells survival [1, 2, 5]. We and others recently found that DEPTOR is a novel substrate of SCF^β-TrCP^ (SKP1, Cullins, F-box proteins) E3 ubiquitin ligase [6–8], the largest family of E3 ubiquitin ligases that regulates many biological processes via degrading the key regulators [9, 10]. Upon growth factor exposure, DEPTOR was phosphorylated at the β-TrCP binding motif (D-pS-G-X-X-pS) on codons 286-291 by RSK1/S6K1 kinases and then recognized by β-TrCP for targeted degradation by SCF E3, leading to mTOR activation [8]. We further showed that by degrading DEPTOR, SCF^β-TrCP^ regulates cell survival [8].

Pancreatic ductal adenocarcinoma (PDAC) is the fourth leading cause of cancer death in the United States and amongst the deadliest human malignancies with an approximate 5 year survival of 5% [11]. Mutational activation of the *Kras* oncogene occurs in 95% of cases, with inactivation of tumor suppressors, p53 and PTEN, and activation of PI3K-AKT-mTOR pathway occurring in ~50-60% of cases [12–16]. In addition, NFκB, a downstream mediator of mutant Kras signaling in PDAC [17, 18], was found to be constitutively activated in most primary pancreatic cancers and cell lines [19, 20], due partly to the overexpression of β-TrCP [21], a F-box protein of SCF E3 ligase that promotes degradation of IκB [22] to activate NFκB and DEPTOR to activate mTORC1/2 [6–8].

Although DEPTOR has been shown to inhibit mTOR activity and may act as a tumor suppressor in some cell culture settings [1, 2], it has not been previously tested whether and how DEPTOR plays a role in pancreatic cancer. Here we show that DEPTOR expression is gradually reduced in pancreatic preneoplastic lesions, and completely lost in 99% of pancreatic ductal adenocarcinoma tissues. Ectopic expression of DEPTOR in pancreatic cancer cells suppressed their growth in monolayer culture and in soft agar, and decreased clonogenic survival by inducing apoptosis and growth arrest, likely through inactivation of mTORC1 signal. Our study suggests that DEPTOR acts as a tumor suppressor in pancreatic cancer and its loss may contribute to the initiation and progression of pancreatic tumorigenesis.

## RESULTS AND DISCUSSION

### DEPTOR expression was lost in PDAC tumor tissues

**Figure 1:**
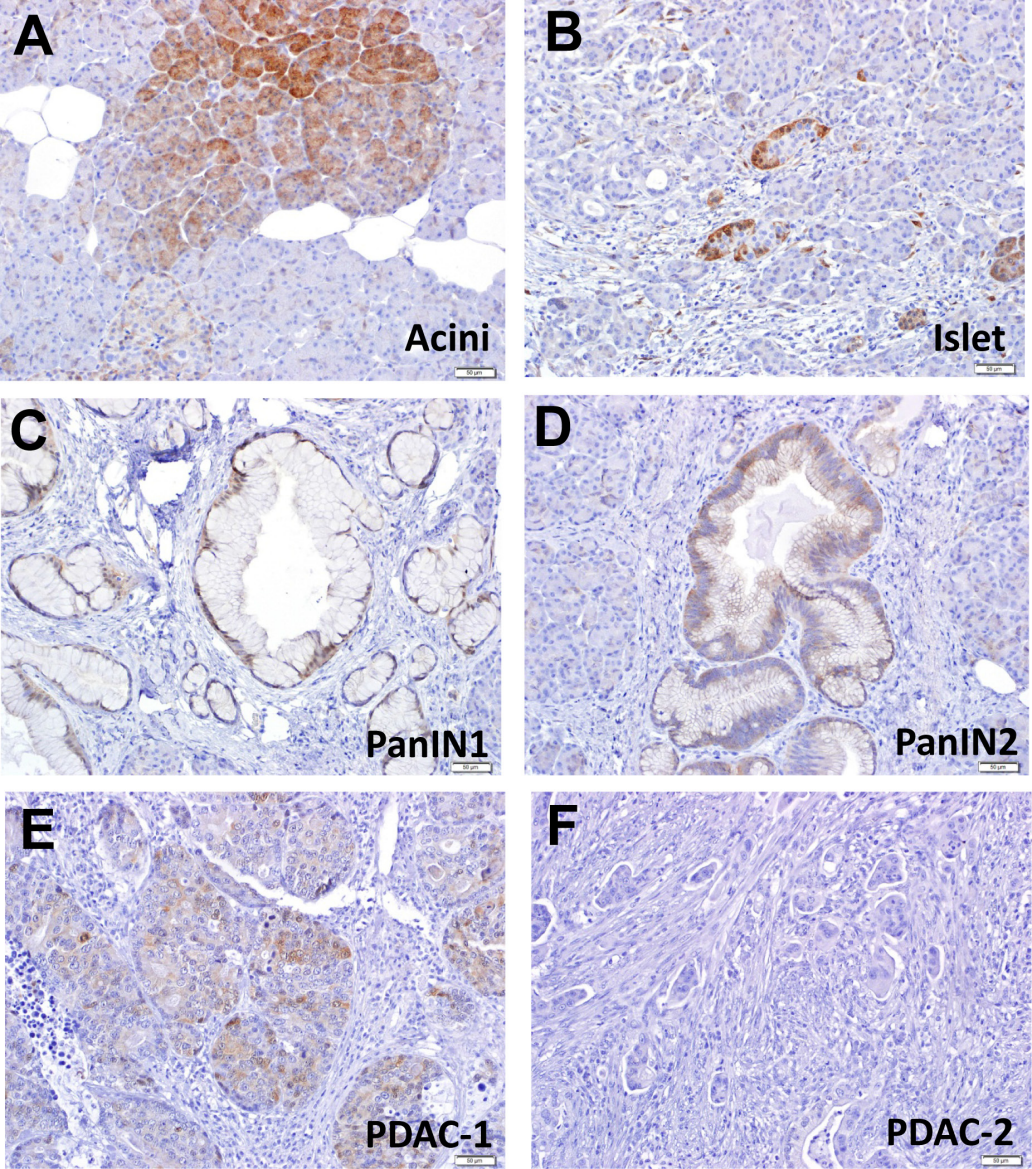
DEPTOR expression is gradually lost during human pancreatic tumorigenesis two sets of PDAC tissue microarrays, containing a total of 101 PDAC tissues, along with 6 normal pancreas tissue slides, were stained with DEPTOR antibody. DEPTOR staining in all samples were scored in blind manner, and photographed. Normal pancreatic cells (**A&B**), preneoplastic lesions (PanIN1, PanIN2) (**C&D**), and PDAC (**E&F**) were labeled as indicated.

To determine potential role of DEPTOR during human pancreatic tumorigenesis, we determined the expression of DEPTOR protein in normal pancreas cells as well as in early pancreatic preneoplastic lesions and PDAC tissues. We found that DEPTOR is expressed in cytoplasm in both normal acinar (Figure 1A) and islet cells (Figure 1B) in a patchy manner. However, DEPTOR expression is gradually decreased from earlier-to-later stage of lesions, such as PanIN1 (Figure 1C), PanIN2 (Figure 1D), one PDAC tissue (Figure 1E). DEPTOR expression is completely lost in 100 out of 101 PDAC tissues (Figure 1F and data not shown). Thus, DEPTOR expression is gradually lost during human pancreatic tumorigenesis.

### Ectopic DEPTOR expression inhibits growth of pancreatic cancer cells in monolayer culture

**Figure 2:**
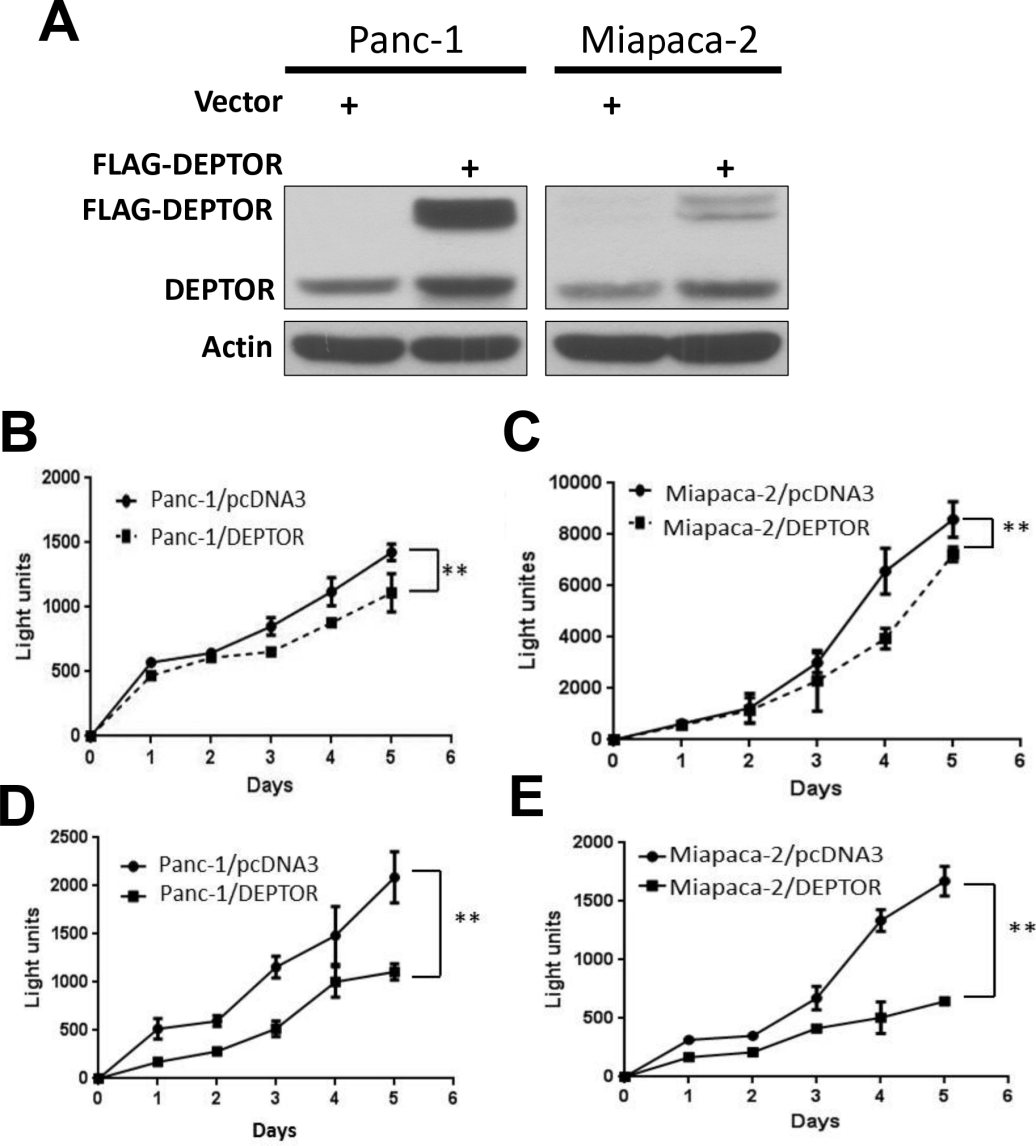
DEPTOR expression inhibits monolayer growth of PDAC cells Panc-1 and Miapaca-2 cells were transfected with mammalian expressing vector encoding FLAG-DEPTOR, along with empty vector pcDNA3 control. Stable clones after G418 selection were pooled and subjected to IB using anti-DEPTOR antibody Ab (**A**). Cells were seeded and cultured in 96-well plates (1500/well) in triplicate for up to 5 days in DMEM with 10% FBS (**B&C**) or DMEM with 1% FBS (**D&E**). Cells were lysed every 24 hrs and subjected to ATP-lite proliferation assay. Shown is X ± SEM of light unit (n = 3). Student *t* test was performed, **, *p* < 0.01.

Given the observation that DEPTOR expression was lost in PDAC tissues, we next determined whether ectopic DEPTOR expression would affect the growth of pancreatic cancer cells. We transfected FLAG-tagged DEPTOR cDNA, along with the empty pcDNA3 control, into two commonly used pancreatic cancer lines, Panc-1 and Miapaca-2. All resistant stable clones, after G418 selection, were pooled for Western blotting. As shown in Figure 2A, the levels of ectopically expressed DEPTOR were comparable to those of the endogenous DEPTOR in both Panc-1 and Miapaca-2 cells. We then measured the growth rate by ATP-lite proliferation assay for a 5-day period and found that in both cell lines, DEPTOR expression inhibited cell growth when cultured in normal medium containing 10% fetal bovine serum (Figure 2B&C). Growth suppression was more substantial if cells were cultured in serum starved condition with 1% FBS (Figure 2D&E). Thus, DEPTOR expression had negative impact on the growth of pancreatic cancer cells, particularly under nutrient-deprived conditions.

### Ectopic DEPTOR expression inhibits clonogenic survival of pancreatic cancer cells

**Figure 3:**
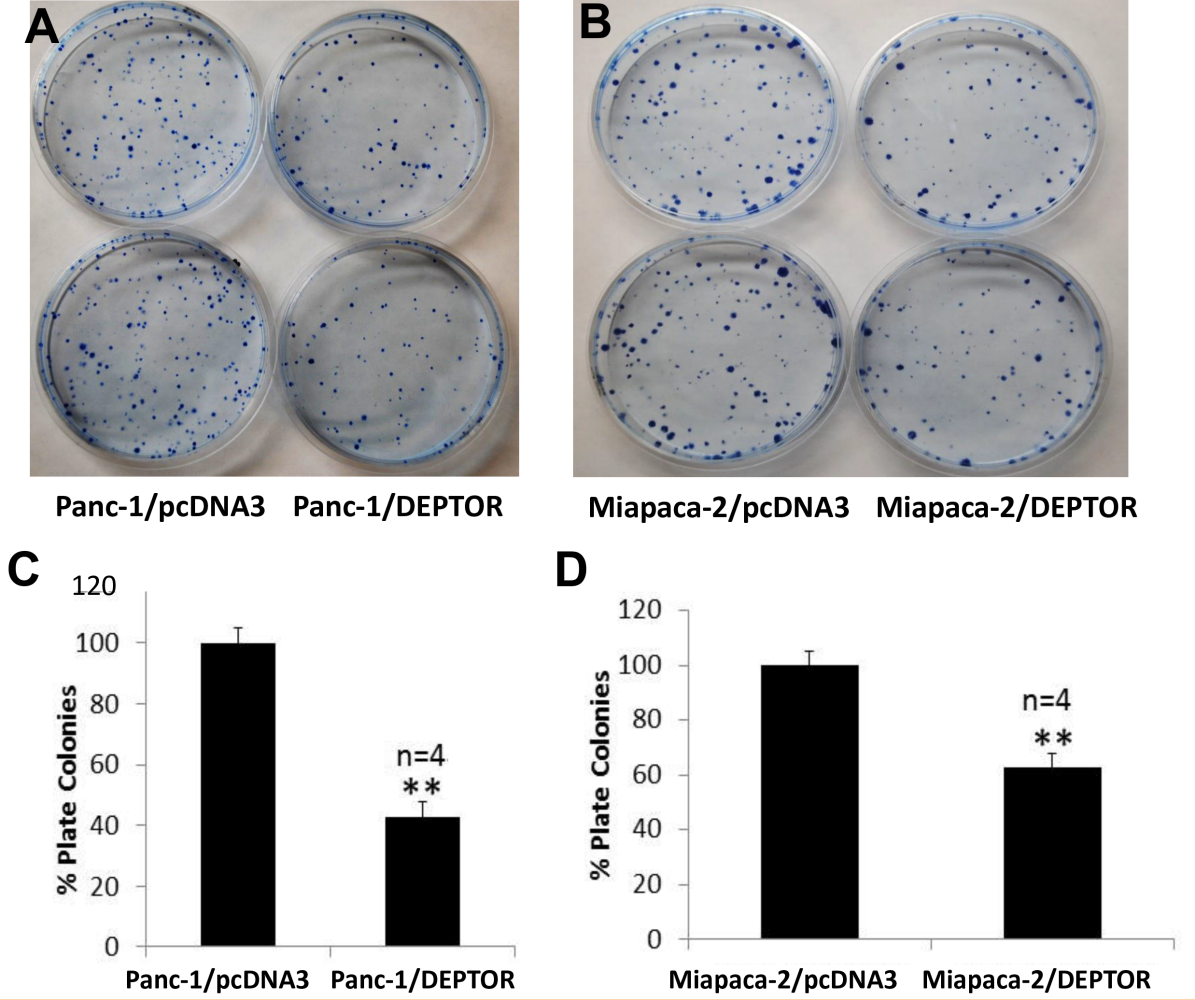
DEPTOR expression inhibits clonogenic survival of PDAC cells Single cell suspension of Panc-1 and Miapaca-2 cells, stably expressing FLAG-DEPTOR were seeded, along with the vector control cells, into 60-mm dishes (600/dish) in DMEM with 10% FBS and grown for 10-14 days. Colonies (≥ 50 cells) were counted and plates were photographed (**A&B**). Quantified results were expressed as percentage to the vector control, setting at 100% (**C&D**). Shown is X±SEM of light unit (n = 4). Student *t* test was performed, **, *p* < 0.01.

We next used a standard clonogenic assay to measure the effect of DEPTOR expression on colony forming ability of these pancreatic cancer cells. Again, colony numbers formed from a single cell were significantly reduced upon ectopic DEPTOR expression in both lines (Figure 3A-D, with representative plates shown in A&B and quantitative data shown in C&D). Thus, DEPTOR expression inhibits the clonogenic survival of pancreatic cancer cells.

### Ectopic DEPTOR expression inhibits anchorage-independent growth of pancreatic cancer cells

**Figure 4:**
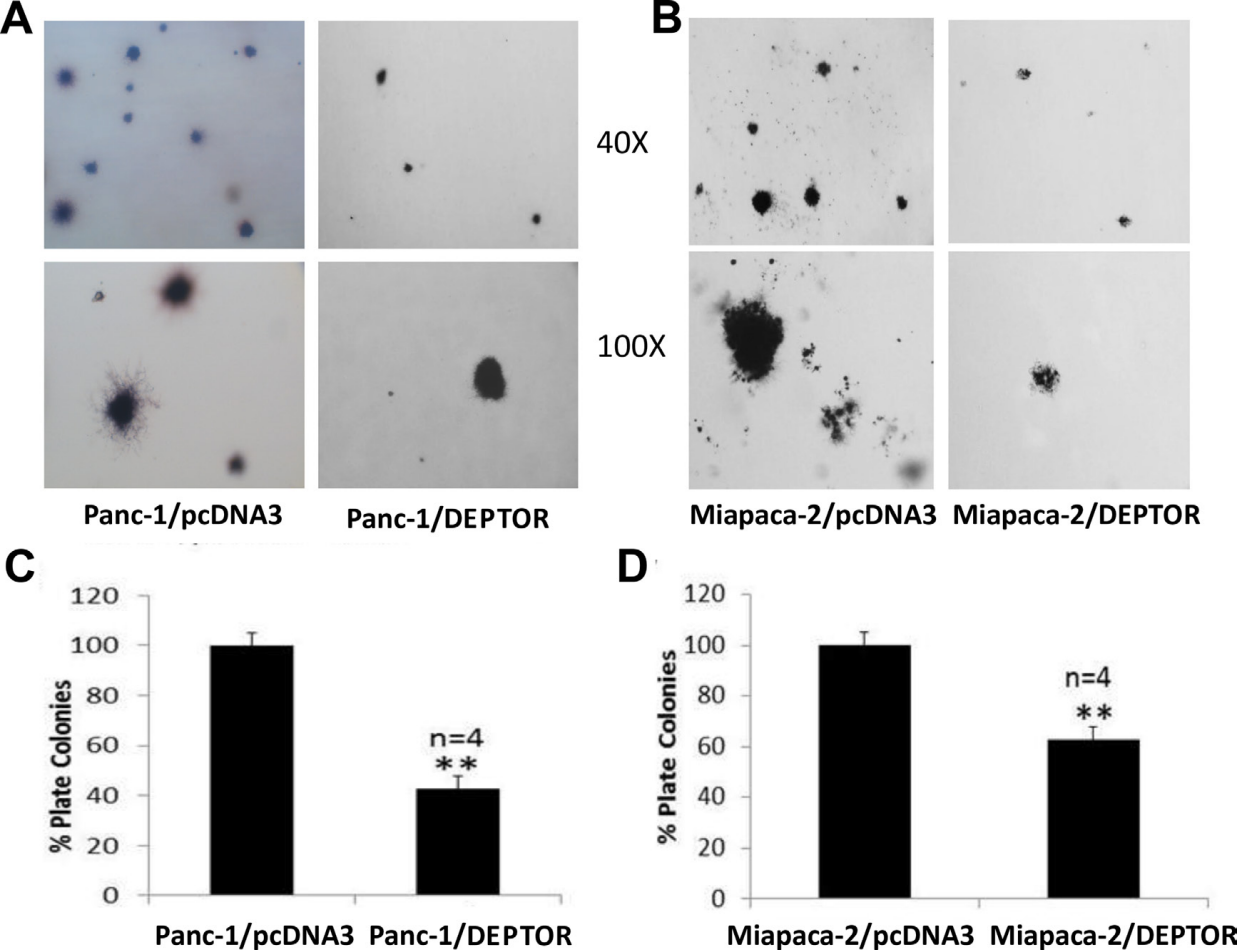
DEPTOR expression inhibits anchorage-independent growth of PDAC cells Single cell suspension of Panc-1 and Miapaca-2 cells, stably expressing FLAG-DEPTOR were seeded, along with the vector control cells, into 60-mm agar dishes (1.5×10^4^ cells/dish), as described [31]. Colonies (≥ 8 cells) were counted after 14 days and photographed (**A&B**). Quantified results were expressed as percentage to the vector control, setting at 100% (**C&D**). Shown is X ± SEM (n = 4). Student *t* test was performed, **, *p* < 0.01.

We further determined the ability of DEPTOR-overexpressing cells to grow in an anchorage independent manner in soft agar, which is a classic measurement of tumor cell phenotype. We found that again, DEPTOR expression significantly suppressed the formation of agar colonies (Figure 4A-D, with representative plates shown in A&B and quantitative data shown in C&D). Taken together, ectopic DEPTOR expression suppressed the growth of pancreatic cancer cells in all measures, indicating a tumor suppressive activity.

### Ectopic DEPTOR expression caused moderate induction of apoptosis and growth arrest

**Figure 5:**
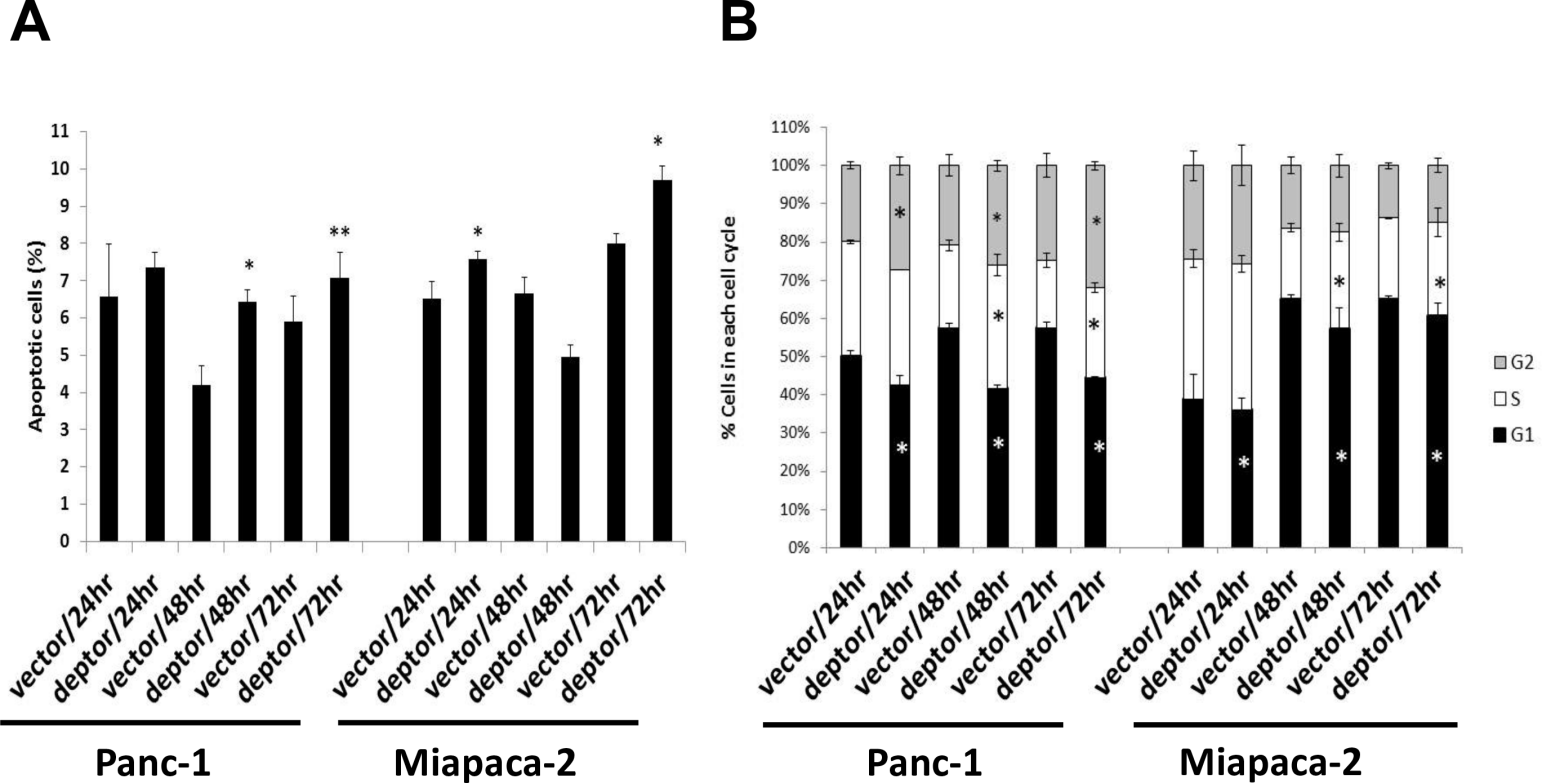
DEPTOR expression moderately induces apoptosis and growth arrest of PDAC cells DEPTOR ectopically expressing Panc-1 and Miapaca-2 cells were seeded, along with the vector control cells, into 60-mm dishes and cultured for up to 72 hrs at DMEM with 10% FBS. Every 24 hrs post seeding, cells were harvested and prepared for FACS analysis. Sub-G1 population was used to calculate % of apoptotic cells (**A**). The remaining population was gated for each phase of cell cycle (**B**). Shown is X ± SEM (n = 2). Student *t* test was performed, *, *p* < 0.05; **, *p* < 0.01.

We then determined the nature of growth suppression using FACS analysis. Cells were grown in monolayer culture for up to 3 days with samples harvested at every 24 hrs. FACS profile revealed that DEPTOR ectopic expression caused a moderate induction of sub-G1 apoptotic population in both cell lines, particularly at later time points (Figure 5A), and also caused growth arrest at the G2/M for Panc-1 cells and the S for Miapaca-2 cells (Figure 5B).

### Ectopic DEPTOR expression inhibits mTORC1 activity

**Figure 6:**
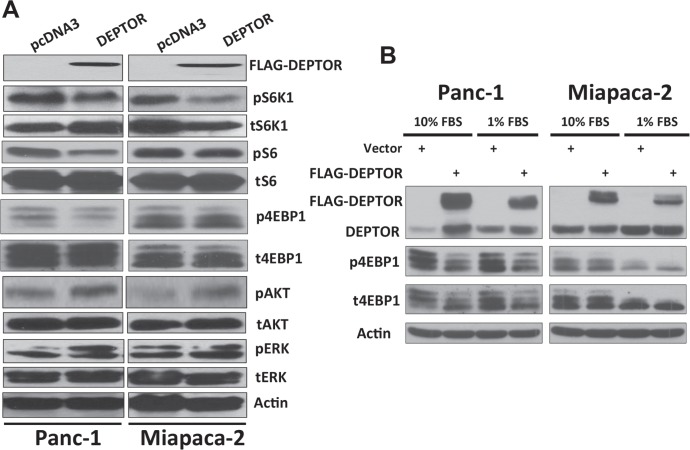
DEPTOR expression inhibits mTORC1 activity in PDAC cells DEPTOR-ectopically expressing Panc-1 and Miapaca-2 cells were seeded, along with the vector control cells, into 60-mm dishes and cultured at DMEM with 10% FBS (**A**) or 10% v.s 1% FBS (**B**) for 24–48 hrs before being lysed for Western blotting analysis using indicated antibodies.

Finally, we evaluated potential mechanisms by which DEPTOR ectopic expression induced growth suppression with a focus on mTORC1 and mTORC2. We found that mTORC1 activity was moderately inhibited upon DEPTOR ectopic expression, as reflected by reduced phosphorylation of mTORC1 substrate S6K and its substrate S6, as well as 4E-BP1, although to a lesser extent (Figure 6A). Interestingly, DEPTOR ectopic expression moderately increased the levels of phosphorylated AKT (Figure 6A), likely due to relieving the feedback inhibition from S6K1 to PI3K, as a result of mTORC1 inactivation [1, 2, 5]. However, DEPTOR ectopic expression had no effect on ERK signal (Figure 6A). Given the fact that growth rate was more severely affected when cells were cultured in 1% FBS condition in DEPTOR transfected cells (Fig. 2B-E), we finally compared mTORC1 activity under growth conditions of 10% FBS vs. 1% FBS. While phosphorylation of S6K was undetectable when both lines of cells were cultured at 1% FBS (data not shown), phosphorylation of 4E-BP1 was indeed more obviously inhibited (Figure 6B).

Several prior studies have reported conflicting correlations between DEPTOR expression and patient prognosis or disease status in human malignancies. One study showed that DEPTOR overexpression correlates with poor prognosis in differentiated thyroid carcinoma [23], whereas another study revealed that DEPTOR expression negatively correlates with tumor progression in colorectal cancer [24]. Here we show that DEPTOR expression is gradually lost during pancreatic tumorigenesis, and completely lost in PDAC tissues. We further showed that ectopic expression of DEPTOR at the level comparable to the endogenous one suppresses the growth of pancreatic cancer cells in monolayer culture, in clonogenic survival and in soft agar likely due to moderate induction of apoptosis and growth arrest at the S or G2/M phases of cell cycle. Interestingly, we found that while DEPTOR overexpression moderately inactivates mTORC1, it activates PI3K/AKT as well, which was also seen in other model systems [8, 25]. The fact that DEPTOR ectopic expression suppresses the growth of pancreatic cancer cells indicates that mTORC1 inactivation out-competes AKT activation. Finally, it is noteworthy that growth suppression by ectopic DEPTOR expression may also be mediated by other yet-to-be identified mechanism(s) in addition to modulation of mTOR pathway. Taken together, our study provides experimental evidence that DEPTOR plays a tumor suppressive role in pancreatic cancer cells and loss of DEPTOR expression could contribute to pancreatic tumorigenesis.

## METHODS

### Cell culture and DNA Transfection

Panc-1 and Miapaca-2 cells were cultured in DMEM supplemented with 10% or 1% fetal bovine serum. Cells were transfected with FLAG-DEPTOR, constructed in pcDNA3 [8], using Lipofactamine 2000. Following G418 selection of 2 weeks, resistant clones were pooled for Western blotting as described [26] for DEPTOR expression, and used for various growth assays. The antibodies were purchased as follows: DEPTOR, Phospho-4EBP1, 4EBP1, Phospho-ERK1/2 (Thr202/Tyr204), ERK1/2, Phospho-S6K1 (Thr389), Phospho-AKT (Ser473), AKT (Cell Signaling Technology, MA), S6K1, β-Actin (Santa Cruz Biotechnology, CA), FLAG (Sigma, MO).

### Immunohistochemical staining

Collection and preparation of pancreatic cancer tissue microarrays containing 101 patient samples were partially described previously [27]. Immunohistochemical staining of the tissue microarrays, along with six normal pancreatic tissues, was performed using the ABC Vectastain kit (Vector Laboratories) with antibody against DEPTOR/DEPDC6, as described [28]. Sections were developed with DAB and counterstained with haematoxylin.

### Cell growth assays

For cell proliferation, an ATPlite kit (Perkin Elmer) was used as described [29] to measure growth rate within 5 days. For cell survival, a standard clonogenic assay was used as described [30]. For measurement of anchorage independent growth, a soft agar assay was used, as described [31].

### Fluorescence activated cell sorting (FACS)

FACS analysis was performed as described [29]. Briefly, cells were harvested and fixed in 70% EtOH at −20°C for at least 4 h, suspended in 1X Propidium Iodide solution with 400 mg/ml RNase (Roche), and analyzed in the Flow Cytometry Lab facility at the University of Michigan. The percent apoptosis is the percent of cells in the subG1 population.
